# The impact of place and legacy framing on climate action: A lifespan approach

**DOI:** 10.1371/journal.pone.0228963

**Published:** 2020-02-25

**Authors:** Robert H. Wickersham, Lisa Zaval, Nancy A. Pachana, Michael A. Smyer

**Affiliations:** 1 Department of Psychology, University of Colorado Colorado Springs, Colorado Springs, Colorado, United States of America; 2 Department of Psychology, Columbia University, New York, New York, United States of America; 3 School of Psychology, The University of Queensland, Brisbane, Australia; 4 Department of Psychology, Bucknell University, Lewisburg, Pennsylvania, United States of America; Middlesex University, UNITED KINGDOM

## Abstract

Despite several decades of research on more effectively communicating climate change to the general public, there is only limited knowledge about how older adults engage with an issue that will shape and define future generations. We focus on two key factors that may motivate younger and older adults to engage in climate change action, legacy concern and place attachment, and assess whether older adults differ in any appreciable way from the general population in this domain. We randomly exposed participants of different ages to either a Legacy, Place, or control writing induction task before they completed various self-report measures. Both induction conditions were associated with significantly greater pro-environmental behavioral intentions and donations for all age groups when compared to the control condition. Legacy motivation and biophilia were used as manipulation checks and found to partially mediate these effects. Findings suggest that legacy and place message framing may be useful in prompting adults of all ages to take action to help combat climate change.

## Introduction

Climate change is real [1,2] and accelerating in its pace and impacts [3,4]. The health and economic impacts of climate change are documented [2,5] with older adults highlighted as a potentially vulnerable group. However, critical psychological barriers to citizen engagement and participation in combating climate change remain [6].

Communication research has explored how subtle behavioral interventions and messaging frames can both increase citizen engagement in climate change issues and encourage pro-environmental behaviors. For example, highlighting local impacts of climate change makes the benefits of acting to mitigate climate change more tangible, leading people to act more sustainably [7]. Another effective strategy is to reduce temporal and psychological distance from the issue by framing climate change as an immediate threat, rather than a remote problem [8]. Messages and intervention strategies targeted to the intrinsic values of a specific audience may be most effective in motivating action [9]. However, one potentially important question has received less attention in the framing literature: how do older adults engage with the issue of climate change?

Older adults, defined here as those beyond child-rearing age, are sometimes left out of climate change discussions, despite representing an untapped and potentially powerful group to assist in changing sustainability behaviors [10]. Older adults are important for developmental and political reasons. Developmentally, older adults are naturally concerned with their legacy and with the well-being of successive generations [11]. Politically, older adults can be potential thought leaders: in developed countries, they vote disproportionately more than other age groups [12]; in traditional societies, they are often looked to as sources of wisdom. They are motivated to make meaningful contributions to society through engaging with their communities [13]. The intersection of older people’s valuing of legacy and the urgent need for positive actions to achieve environmental sustainability could be a pathway for intergenerational collaboration and progress on these urgent issues.

However, research is needed to advance an understanding of not only how age differences in sustainability behaviors emerge, but also how the adoption and implementation of interventions to promote sustainability among older adults can best be achieved. The effect of chronological age on sustainability behaviors may depend on several psychological factors that differ between individuals and change across the lifespan. Theoretical insights from relevant psychological and gerontological scholarship suggest two central factors that stand out: place attachment and legacy concern.

Place attachment, the formation of emotional and cognitive bonds with a particular place [14], may have particularly strong implications for older adults. Place attachment includes both the psychological process of attaching and the product of that process [15]. Older adults have had greater time to forge attachments to place, and also have experienced changes to places of personal significance over their lifespans. Some suggest that older adults’ attachment to place is linked to their continuity of self across the life-span [16]. More recent work confirms the strong, positive link between age and place attachment [17,18].

This study advances knowledge about place attachment in the context of sustainability behaviors. Devine-Wright (2013) [19] urged more attention to the links between place attachment and climate change, especially the impact of framing place-based climate communication to engage the public. There have been initial attempts to explore the impact of place-based communication strategies [20]. Thus far, however, the impact of older adults’ attachment to place on their sustainability behaviors has not been investigated.

Both place attachment and concern about climate change may be related to biophilia, humans’ particular affiliation and emotional connection to the natural world [21]. Climate change threatens many aspects of the natural world (as well as the built environment), and the places that people are most attached to are often inexorably linked to nature. Research on the salience of older adults’ biophilia includes everything from its impact on well-being and participation in activities in the natural world such as gardening [22] and driving through natural areas [23] to the potential public health benefits of exposure to the natural environment [24]. However, biophilia has not previously been considered as a mediational pathway for sustainability behaviors in the context of older adults’ attachment to place.

In addition to place attachment, another psychological variable with strong implications for older adults’ environmental behavior is their motivation to leave behind a positive legacy for future generations. Environmental protection clearly involves a temporal element, securing natural resources for future generations. The meaning of this long time frame may shift with increasing age, as constraints on time left in life may change priorities about how to use the remaining time. As people age, generative concerns, defined as a person’s desire to guide future generations, become increasingly significant [25]. Generative and legacy concerns are strongly correlated with pro-environmental action, and inducing people to reflect upon their future legacy can promote pro-environmental engagement [26]. We predict that older adults’ concern for their legacy should promote pro-environmental action, since seniors may be particularly motivated to leave a positive legacy and take responsibility for future generations. Interventions and message frames designed to increase legacy motives may therefore be particularly powerful among older adults.

Since older adults have higher levels of legacy concern and place attachment, leveraging these psychological motives could serve as effective intervention strategies to promote environmental behavior among older adults. The current study assesses the impact of two motivational strategies—an induction designed to increase legacy motivation and an induction designed to enhance place attachment—on pro-environmental behavioral intent and behavior (donations to an environmental charity). Specifically, this study has three goals: (1) to replicate and expand upon the findings of an earlier study on the impact of legacy motives on pro-environmental behaviors [26] by exploring whether legacy motivations can be leveraged among an older adult sample; (2) to provide initial information on the impact of a place-based induction [27] on climate change behaviors across the lifespan; and (3) to assess the mediating impacts of individual differences on variables linked to aging and sustainability behaviors.

Within this context, we have three hypotheses:

1. That the Legacy induction will be positively associated with pro-environmental behavioral intentions and pro-environmental behavior;
2. That the Place induction will be positively associated with pro-environmental behavioral intentions and pro-environmental behavior;
3. That older adults exposed to the Legacy and Place inductions will experience greater positive associations with pro-environmental intentions and behaviors than younger adults.

## Materials and methods

### Participants

A diverse sample of 1005 American participants were recruited and completed our study between 12/7/16 and 12/27/16 using the advanced sampling procedures available through Amazon’s TurkPrime (an enhanced version of Amazon’s mTurk) to achieve an even distribution across ages in the sample, ranging from 18–87. Specifically, TurkPrime’s HyperBatch, IP tracking, and age-based quota features were used to simultaneously recruit 5 groups of 200 American adults from the following age groups: 18–30, 31–40, 41–50, 51–60, and 61+ (64–87). Online experiment services like mTurk have numerous advantages and have been found to be at least as valid as traditional recruitment methods [28]. All participants were given the opportunity to review study details and informed consent information via mTurk before electing to participate in the experiment and following a hyperlink to begin our online survey, which was hosted by Qualtrics. This experiment was approved by the University of Colorado Colorado Springs Institutional Review Board.

Five attention checks were used as quality control measures and were included in the experimental design, with an a priori cutoff of missing three or more attention checks, or blatantly not following instructions, set as the criteria for exclusion from analysis. One attention check gave lengthy instructions but asked participants to simply write “I read the instructions” and to ignore the multiple choice options presented on the page. Other attention checks involved assessing timestamps and screening for nonsensical responses on the writing task to see if participants complied with instructions, or simply clicked through the experiment as quickly as possible. Based on these criteria, 17 participants were excluded from analysis, leaving a remaining total sample of 988. Because this study was able to achieve an even distribution of ages across the sample, participants were then divided chronologically into three age groups for analysis: young adults 18–35 (*n* = 327), middle-aged adults 36–52 (*n* = 324), and older adults 53–87 (*n* = 337). An estimate of target sample size was determined using results from similar studies run using mTurk workers to provide initial estimates of effect sizes for studies of this type, which suggested a small to medium effect size [29]. Thus, our final sample size of 988 participants (with about 330 for each age group) was deemed appropriate for our planned experimental design.

An analysis of demographic information provided by participants revealed that 68% of our sample identified as female (*n* = 670), 32% identified as male (*n* = 316), and .2% identified as ‘other’ (*n* = 2). When asked about the highest level of education completed, .5% reported some high school (*n* = 5), 10% reported graduating high school (*n* = 100), 34% reported some college or technical school (*n* = 338), 37% reported graduating college or technical school (*n* = 369), and 18% reported receiving post-graduate education (*n* = 176). Politically, our sample was well divided, with 28% identifying as Republicans (*n* = 277), 39% as Democrats (*n* = 384), 28% as Independents (*n* = 272), and 5% identifying as ‘Other’ (*n* = 55). Participants were able to select as many ethnicities/races they identified with, and thus a total of 86% identified as Caucasian (*n* = 849), 9% as African American (*n* = 93), 5% as Hispanic (*n* = 45), 5% as Asian (*n* = 45), 3% as Native American (*n* = 29), 1% as Other (*n* = 10), and .1% as Pacific Islander (*n* = 1). Finally, when asked whether participants identified as environmentalists, 55% said no (*n* = 546) and 45% said yes (*n* = 441).

### Measures

Two measures were used as dependent variables, one was a scale measuring pro-environmental behavioral intentions, and one was a proxy for pro-environmental behavior. The Pro-Environmental Behavioral Intentions Scale [26] is a 6-level likert scale with six items measuring the participant’s intent to make pro-environmental choices in the next month (Range = 0–6, *M* = 2.73, *SD* = 0.85, α = .76; e.g., “over the next month, how often do you intend to use public transportation or carpool?”). Following Zaval et al. (2015) [26], the second measure served as a proxy for real pro-environmental behavior, an open numerical item informing participants that they had been entered into a $10 dollar lottery, which asked how much of that money they would donate to the charity Trees for the Future if they won.

Two measures were used as manipulation checks to ensure that each induction was evoking the responses intended. The Legacy Motive Scale [26] was used as a manipulation check for the Legacy induction. This 8-item scale measures how much participants care about their legacy (Range = 0–6, *M* = 4.19, *SD* = 1.05, α = .90; e.g., “I care about what future generations think about me”). The 5-item Abbreviated Biophilia Scale was used to measure participants’ love and appreciation for nature, and was used as a manipulation check for the Place induction (Range = 0–7, *M* = 5.36, *SD* = 1.10, α = .97; e.g., “I feel joy just being in nature”; adapted from Perkins, 2010 [30]).

Participants also provided extensive psychographic data that could potentially mediate the relationship between the inductions and environmental action. Two hazard belief questions were adapted from Miceli, Sotgiu, & Settanni (2008) [31], these 4-level likert questions (Range = 0–4), asked about the participant’s belief in the connection between natural hazards, climate change, and salient danger. A single-item measure of future orientation developed by Steinberg et al. (2015) [32] was included and asked participants to describe on a scale of 1–10 how easily life in the year 2050 comes to mind. An assessment of Future Self-Continuity was included asking participants to choose one picture (of seven available pictures) of two overlapping circles (each picture overlapping to increasingly higher degrees) best representing how connected they felt to their future selves [33]. A single-item measure of internal locus of control asked participants to rate how much control they had over their fate from (low internal control)1-10 (high internal control) [34]. A measure of mortality salience, the Death Thought Accessibility Word Completion Task, was used to explore whether either induction was evoking thoughts of death and whether this was related to observed outcomes [35]. Finally, demographics questions assessing age, gender, education, political affiliation, ethnicity, and whether participants identified as environmentalists were included. Methodological details including full versions of all scales, measures, and attention checks used in this study are provided in S1 File.

### Procedure

Participants who met sampling requirements viewed an mTurk advertisement for a 20–35 minute survey and writing task. Once participants provided informed consent and opted to participate in the study, they were randomly exposed to one of three different 5–7 minute writing tasks, representing three induction conditions—Place (*n* = 299), Legacy (*n* = 295), and Control (*n* = 394).

Writing task one, the Place-based induction (Place), asked each participant to write about a place that he or she had an emotional connection to, and to imagine how extreme weather events or climate change might impact that place and change it in the future. This induction was adapted from Smyer (2017) [27]. Writing task two, the Legacy induction (Legacy), was adapted from Zaval et al. (2015) [26], and asked participants to write about what they wanted future generations to remember them for, and in what ways they will have a positive impact on the environment or other people after they are gone. Writing task three, the control induction, asked participants to write detailed instructions on a topic unrelated to place, climate change or legacy: they were asked to write a description of how to make scrambled eggs. Complete induction prompts are provided in S2 File.

Following exposure to their randomly assigned writing task, all participants completed demographic questions and our full battery of self-report measures and scales. After completing all measures and questions, participants entered a code and were digitally thanked, paid, and debriefed. Once a sufficient number of participants had completed the online experiment, data collection was closed, and collected data were de-identified and downloaded for analysis.

Our analytical approach was to conduct a series of 3 (Induction) x 3 (Age) factorial ANOVAs. The independent variable ‘Induction’ had 3 levels: Legacy, Place, and Control. The independent variable ‘Age’ also had 3 levels: young adults 18–35, middle-aged adults 36–52, and older adults 53–87. 3x3 Factorial ANOVAs were run for each dependent variable: one for mean pro-environmental intention score, and one for the amount participants volunteered to donate to planting trees. We then conducted mediation analyses using two measures we had included as manipulation checks, legacy motivation and biophilia attitudes, to assess whether they mediated the relationships between our inductions and our dependent variables. Finally, several exploratory 3x3 Factorial ANOVAs were run using the rest of the psychographic scales included in our testing battery, to identify variable relationships underlying observed effects.

## Results

### Statistical assumptions

To ensure statistical assumptions necessary for a series of 3 (Induction) x 3 (Age) factorial ANOVAs had been met, data were screened for outliers, homogeneity of variance, and univariate normality. Histograms were visually inspected for outliers, with none identified. In all cases, Levene’s test was satisfied or standard deviation ratios did not exceed 2:1, suggesting adequate homogeneity of variance between groups. Additionally, Bartlett’s test indicated sphericity assumptions were adequately met or corrected for in all analyses.

### Manipulation checks

#### Legacy motivation

**Fig 1:**
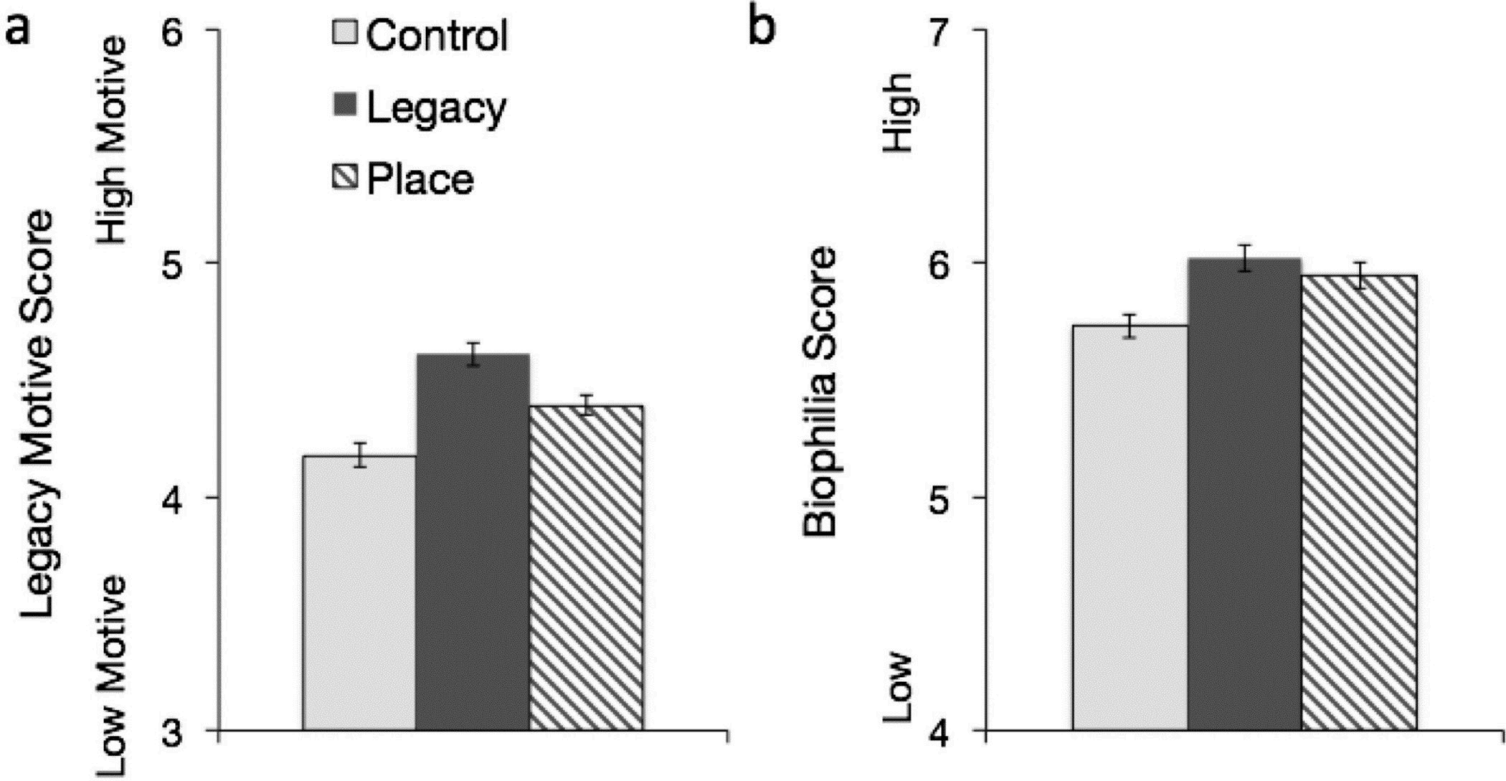
Impact of legacy and place inductions on legacy motives and biophilia scores. (a) Impact of Legacy and Place inductions on legacy motives. (b) Impact of Legacy and Place inductions on biophilia scores. Error bars denote ±1 *SEM*. Control *n* = 394, Legacy *n* = 295, Place *n* = 299.

As expected, the Legacy induction was associated with greater legacy motivation (Fig 1A). A significant main effect emerged for induction *F* (2, 967) = 20.62, *p* < .001, *η*^*2*^ *=* .041, such that Place (*p* = .001) and Legacy (*p* < .001) inductions were associated with greater legacy motivation compared to the control condition. Additionally, the Legacy induction was associated with significantly greater legacy motivation than the Place induction (*p* = .002). In line with previous literature, a significant main effect also emerged for age on legacy motivation *F* (2, 967) = 3.84, *p* = .022, *η*^*2*^ *=* .008, such that young adults had significantly less legacy motivation than middle-aged adults or older adults, while middle-aged and older adults did not differ significantly. No significant interaction effect between age and induction on legacy motivations emerged (*p* = .793).

#### Biophilia attitudes

As predicted, the Place induction was associated with greater feelings of biophilia (Fig 1B). A significant main effect emerged for induction *F* (2, 979) = 8.12, *p* < .001, η^*2*^ *=* .016, such that Place (*p* = .002) and Legacy (*p* < .001) inductions were associated with greater biophilia compared to the control condition, while the Place and Legacy inductions did not differ significantly from each other. Consistent with previous literature [36], greater biophilia overall correlated with greater chronological age across the sample. A significant main effect emerged for age on biophilia *F* (2, 979) = 20.78, *p* < .001, *η*^*2*^ *=* .041, such that older adults reported significantly greater biophilia than middle-aged adults, and middle-aged adults reported significantly greater biophilia than young adults. However, no significant interaction effect between age and induction on biophilia emerged (*p* = .855): the impact of the three inductions on biophilia was similar across all age groups.

### Pro-environmental behavioral intentions

**Fig 2:**
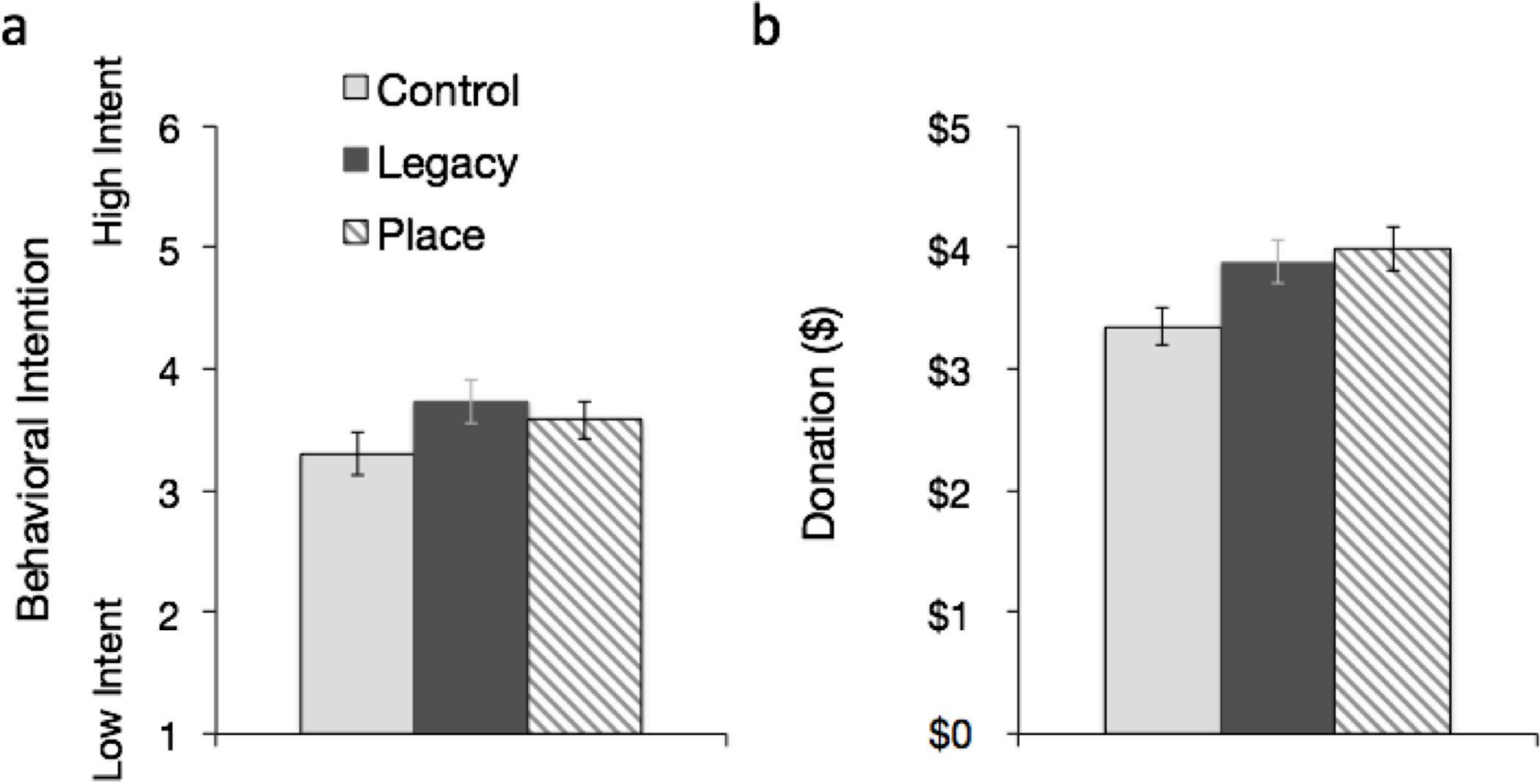
Impact of legacy and place inductions on pro-environmental behavioral intentions and donations to charity. (a) Impact of Legacy and Place inductions on pro-environmental behavioral intentions. (b) Impact of Legacy and Place inductions on donations to an environmental charity. Error bars denote ±1 *SEM*. Control *n* = 394, Legacy *n* = 295, Place *n* = 299.

While older adults had significantly more pro-environmental intentions than middle or young adults overall, both inductions were associated with significantly more pro-environmental behavioral intentions for all ages when compared to the control induction (Table 1 and Fig 2A). These results support hypothesis 1 and hypothesis 2, that the inductions would be positively associated with pro-environmental behavioral intentions, and are contrary to hypothesis 3, that older adults exposed to the inductions would experience greater positive associations with pro-environmental intentions and behaviors than younger adults. A significant main effect emerged for induction *F* (2, 967) = 15.04, *p* < .001, *η*^*2*^ *=* .030, such that Place (*p* < .001) and Legacy (*p* < .001) inductions were associated with more pro-environmental behavioral intentions compared to the control condition, while the Place and Legacy inductions did not differ significantly from each other. A significant main effect also emerged for age on pro-environmental behavioral intentions *F* (2, 967) = 5.89, *p* = .003, *η*^*2*^ *=* .012, such that older adults had significantly greater intentions than middle-aged adults or young adults, while middle-aged and young adults did not differ significantly. However, no significant interaction effect between age and induction on behavioral intentions emerged (*p* = .299). That is, the impact of the three inductions on behavioral intentions was similar across all age groups.

**Table 1:** Mean pro-environmental behavioral intentions by age group and induction type.

Induction	Age Group	Mean	95% Confidence Interval	
			Lower Bound	Upper Bound
Place	Young	3.55	3.34	3.76
	Middle	3.35	3.14	3.56
	Older	3.85	3.65	4.04
Legacy	Young	3.69	3.48	3.90
	Middle	3.64	3.44	3.83
	Older	3.89	3.67	4.10
Control	Young	3.31	3.14	3.48
	Middle	3.27	3.08	3.46
	Older	3.35	3.17	3.52

### Donations to an environmental charity

Both the Place and Legacy inductions were associated with more pro-environmental donations for all ages compared to the control (Table 2 and Fig 2B). These results support hypothesis 1 and hypothesis 2, that the inductions would be positively associated with pro-environmental behavior, and are contrary to hypothesis 3, that older adults exposed to the inductions would experience greater positive associations with pro-environmental intentions and behaviors than younger adults. A significant main effect emerged for induction *F* (2, 979) = 4.30, *p* = .014, *η*^*2*^ *=* .009, such that Place (*p* = .006) and Legacy (*p* = .027) inductions were associated with more donations compared to the control condition, while the Place and Legacy inductions did not differ significantly from each other. A significant main effect also emerged for age on donations, *F* (2, 979) = 5.15, *p* = .006, *η*^*2*^ *=* .010, such that young adults donated significantly less than middle-aged adults or older adults, while middle-aged and older adults did not differ significantly. However, no significant interaction effect between age and induction on donation emerged (*p* = .478).

**Table 2:** Mean donation amount by age group and induction type.

Induction	Age Group	Mean	95% Confidence Interval	
			Lower Bound	Upper Bound
Place	Young	3.58	2.95	4.21
	Middle	4.29	3.67	4.91
	Older	4.09	3.51	4.67
Legacy	Young	3.52	2.89	4.15
	Middle	3.61	3.04	4.19
	Older	4.54	3.91	5.18
Control	Young	2.90	2.38	3.41
	Middle	3.45	2.90	4.01
	Older	3.71	3.19	4.23

### Mediation analyses

**Fig 3:**
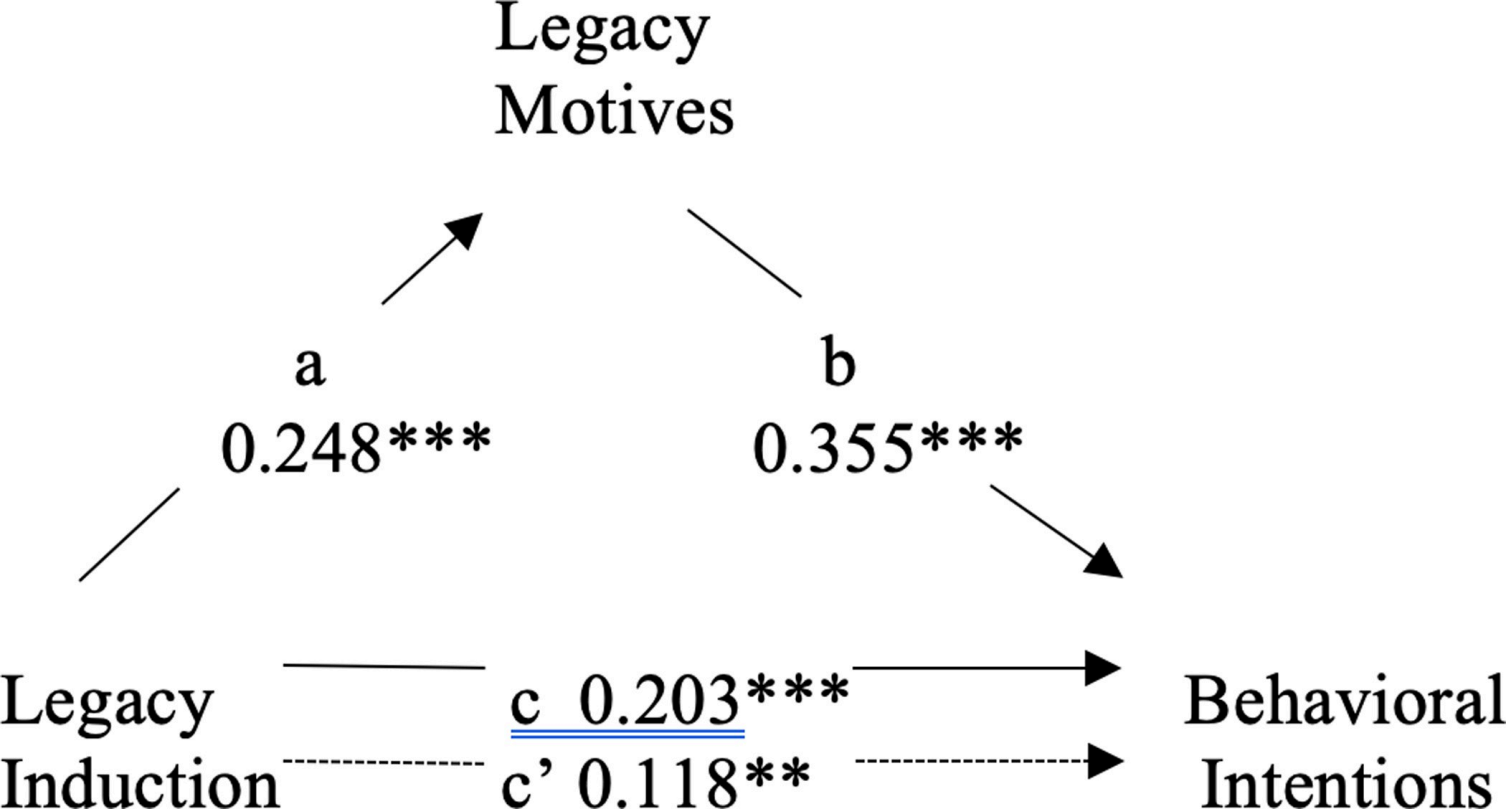
Mediation of legacy induction and behavioral intentions by legacy motives. ***p < .001. **p < .01. *p < .05.

**Fig 4:**
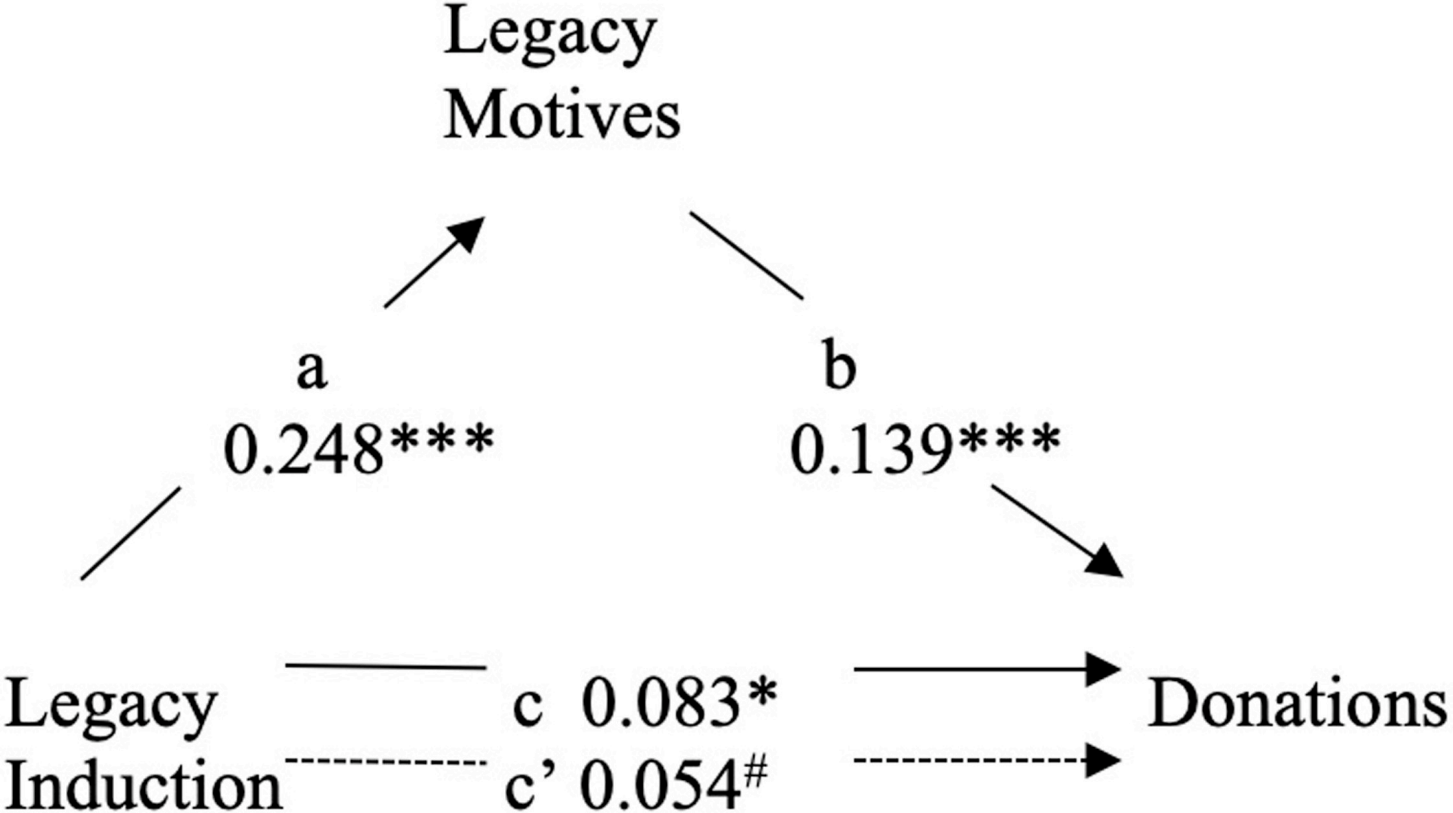
Mediation of legacy induction and donations by legacy motives. ***p < .001. *p < .05. # *p* = .171.

We conducted two mediation analyses to assess whether legacy motivation mediated the Legacy induction’s effect on behavioral intentions (Fig 3), and whether legacy motivation mediated the Legacy induction’s effect on donations (Fig 4). Dummy coding was used on predictor variables to allow for comparisons between the control and Legacy induction conditions. Given the simplicity of our models and the distributions of the variables involved, we chose the straightforward four-step mediation approach of Baron and Kenny (1986) [37]. In step 1 of the first analysis, we revealed that exposure to the Legacy induction had a significant effect on behavioral intentions, *b* = .203, *t*(676) = 5.39, *p* < .001. Next, the Legacy induction was found to have a significant effect on legacy motives, *b* = .248, *t*(675) = 6.64, *p* < .001. In step 3, legacy motives were revealed to have a significant effect on behavioral intentions when controlling for exposure to the Legacy induction, *b* = .355, *t*(665) = 9.67, *p* < .001. Step 4 revealed that the effect of the Legacy induction on individuals’ behavioral intentions was partially mediated by increases in legacy motives: *b* = .118, *t*(665) = 3.22, *p* = .001. Mediation was also confirmed by a bias-corrected bootstrapping procedure using 10,000 samples [38] (Hayes, 2013). This analysis showed that the indirect effect of the Legacy induction on behavioral intentions through legacy motivation was significant, with a 95% confidence interval (CI) that excluded zero (β = 0.180, 95% CI = [0.120, 0.245]).

In step 1 of the second analysis, we revealed that exposure to the Legacy induction had a significant effect on donations, *b* = .083, *t*(685) = 2.19, *p* = .029. Step 2 confirmed that the Legacy induction had a significant effect on legacy motives, *b* = .248, *t*(675) = 6.64, *p* < .001. In step 3, legacy motives were revealed to have a significant effect on donations when controlling for exposure to the legacy induction, *b* = .139, *t*(674) = 3.53, *p* < .001. The final step revealed that the effect of the Legacy induction on donations was fully mediated by increases in legacy motives: *b* = .054, *t*(674) = 1.37, *p* = .171. Mediation was confirmed by a bias-corrected bootstrapping procedure using 10,000 samples (Hayes, 2013). This analysis showed that the indirect effect of the Legacy induction on donations through legacy motivation was significant, with a 95% confidence interval (CI) that excluded zero (β = 0.069, 95% CI = [0.028, 0.118]).

**Fig 5:**
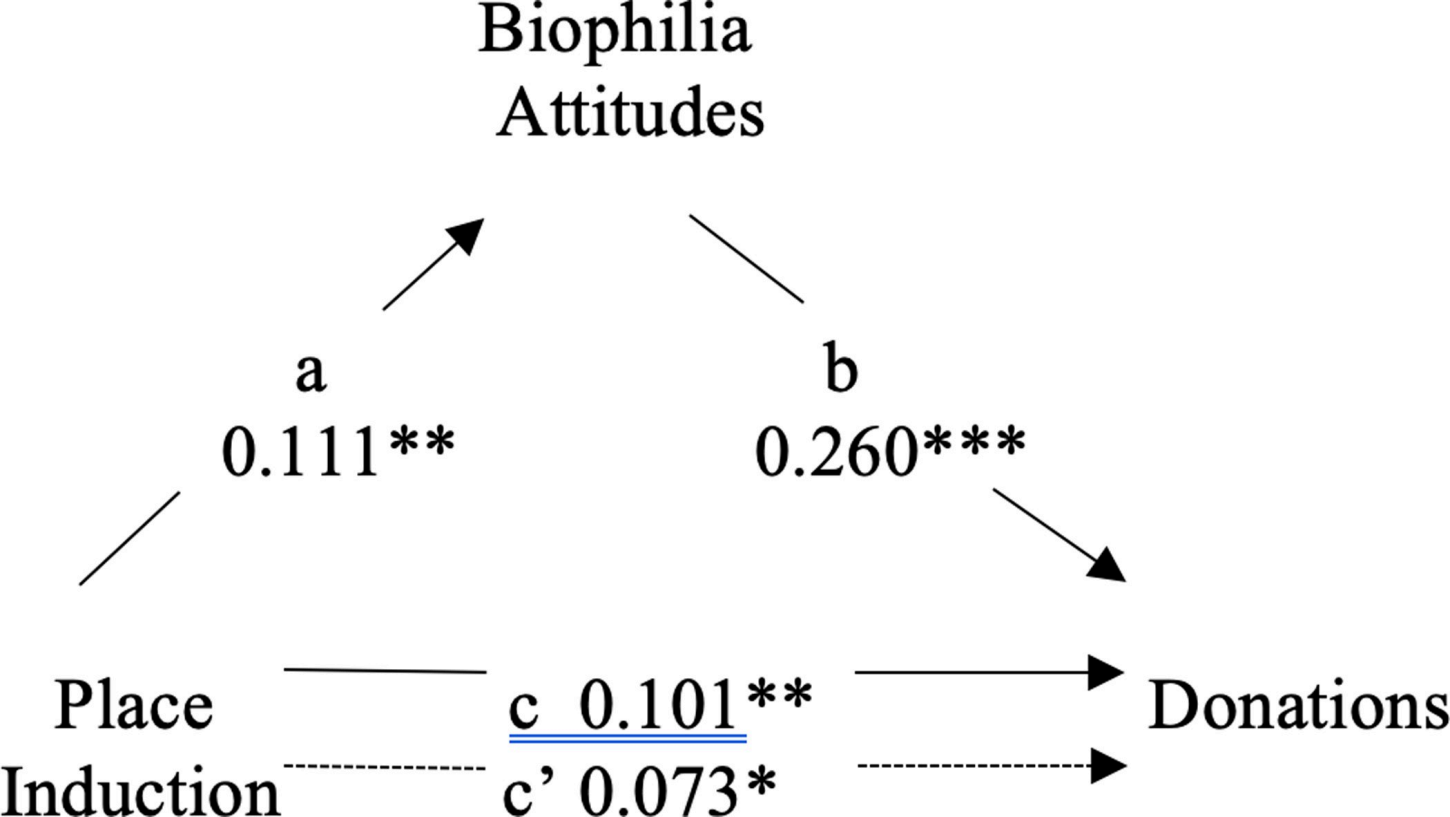
Mediation of place induction and donations by biophilia attitudes. ***p < .001. **p < .01. *p < .05.

**Fig 6:**
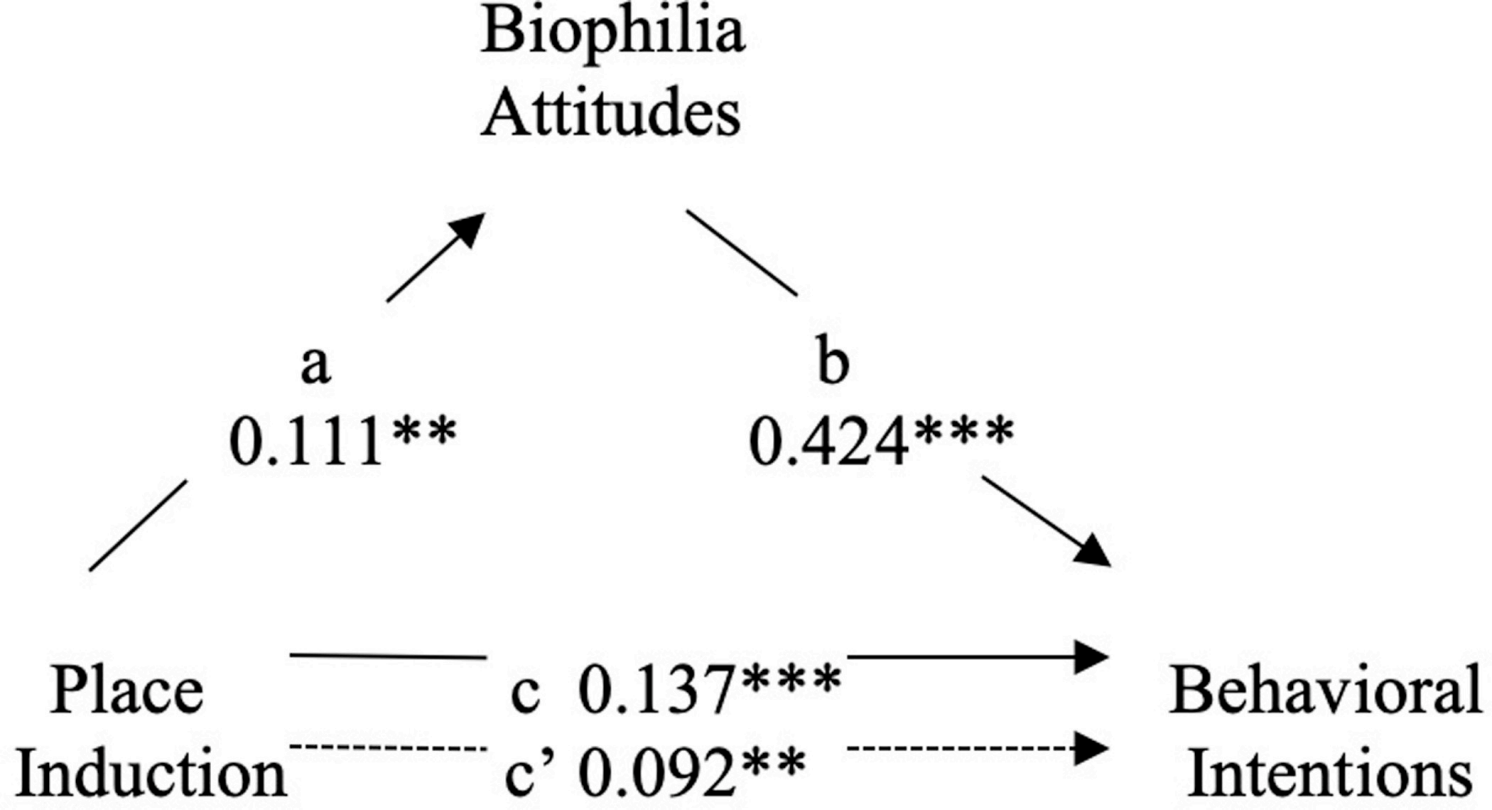
Mediation of place induction and behavioral intentions by biophilia attitudes. ***p < .001. **p < .01. *p < .05.

We conducted two additional mediation analyses to assess whether biophilia mediated the Place induction’s effect on donations (Fig 5), and whether biophilia mediated the Place induction’s effect on behavioral intentions (Fig 6). Dummy coding was used on predictor variables to allow for comparisons between the control and Place induction conditions. In step 1 of the first analysis, we confirmed that exposure to the Place induction had a significant effect on donations, *b* = .101, *t*(690) = 2.68, *p* = .008. Step 2 revealed that the Place induction was found to have a significant effect on biophilia attitudes, *b* = .111, *t*(690) = 2.93, *p* = .004. In step 3, biophilia attitudes were revealed to have a significant effect on donations when controlling for exposure to the Place induction, *b* = .260, *t*(689) = 7.06, *p* < .001. Step 4 revealed that the effect of the Place induction on donations was partially mediated by increases in biophilia attitudes: *b* = .073, *t*(689) = 1.97, *p* = .049. Mediation was again confirmed by a bias-corrected bootstrapping procedure using 10,000 samples [38] (Hayes, 2013). This analysis showed that the indirect effect of the Place induction on donations through biophilia was significant, with a 95% confidence interval (CI) that excluded zero (β = 0.058, 95% CI = [0.018, 0.100]).

We also assessed whether greater biophilia would mediate the effect of the Place induction on behavioral intentions. In step 1 of this analysis, we revealed that exposure to the Place induction had a significant effect on behavioral intentions, *b* = .137, *t*(681) = 3.62, *p* < .001. In step 2, the Place induction was found to have a significant effect on biophilia attitudes, *b* = .111, *t*(690) = 2.93, *p* = .004. In step 3, biophilia attitudes were revealed to have a significant effect on behavioral intentions when controlling for exposure to the Place induction, *b* = .424, *t*(680) = 12.26, *p* < .001. Step 4 revealed that the effect of the Place induction on individuals’ behavioral intentions was partially mediated by increases in biophilia attitudes: *b* = .092, *t*(680) = 2.65, *p* = .008. Mediation was again confirmed by a bias-corrected bootstrapping procedure using 10,000 samples (Hayes, 2013). This analysis showed that the indirect effect of the Place induction on behavioral intentions through biophilia was significant, with a 95% confidence interval (CI) that excluded zero (β = 0.093, 95% CI = [0.028, 0.160]).

To further explore the relationships among these variables, four additional parallel mediation analyses including both mediators simultaneously were conducted and are available in the supplementary material (S3 File).

### Non-significant results

Several other psychological variables that could potentially underlie the relationship between the inductions and pro-environmental behaviors were included in our analysis, including natural hazard perceptions, perceived future self-continuity, and death thought accessibility. However, neither the Place nor Legacy inductions had an effect on these variables (S4 File).

## Discussion

Two elements provide a context for this study: population aging and increased scrutiny of the impact of message framing on pro-environmental behaviors and intention to act. The United States is an aging country in an aging world. Currently, there are 65 million Americans 60 and older, estimated to grow to 100 million by mid-century. Worldwide, the figures are 1 billion now; estimated to be 2 billion by mid-century [39]. At the same time, climate communication specialists are increasingly focused on message framing’s impact on climate action. Thus far, however, climate communicators have not assessed whether specific messages targeted to older adults will have a differential impact on climate concern and intention to act.

Within this context, this study had several goals. The first was to replicate and expand upon prior work on the positive impact of legacy motives on environmental attitude and behavior by exploring whether the impact of a Legacy induction is moderated by age [26]. The second was to provide initial information on the impact of a Place-based induction on environmental attitude and behavior and to explore whether the impact of a Place-based induction is moderated by age [27]. Using messaging to enhance legacy and place attachment is consistent with the National Academies of Science’s (2011) climate communication suggestions to personalize climate change and sustainability issues [40].

Using the MTurk platform, we assembled a balanced sample across three age groups: young adults, middle-aged adults, and older adults. Within this sample, both Legacy and Place inductions were successful at improving pro-environmental attitudes and behavior. Specifically, Zaval and colleague’s (2015) [26] earlier findings on the positive association of a Legacy induction with both pro-environmental behavioral intent and behavior (donations to an environmental charity) were replicated with a balanced age distribution sample. In addition, our results show that an induction aimed at improving attachment to place can positively impact pro-environmental attitude and behavior change across the lifespan, with implications for developing new motivational message frames. Counter to our predictions that the inductions would be most effective for older adults, we observed no age by induction interaction effects. That is, the inductions worked equally well for older and younger adults in promoting environmental action.

The inclusion of a biophilia measure adds an innovative dimension to our understanding of changes in environmental attitude, as we found that both experimental inductions were associated with greater biophilia, and that greater biophilia partially mediated the effect of the Place induction on climate action, across age groups. It may be that increasing love of nature is an underlying mechanism for the effectiveness of place-based framing inductions on behavioral intent and environmental behavior. Additionally, there was a significant correlation between age and biophilia, with greater age associated with greater levels of biophilia. This is an area of communication research that warrants further study.

Both induction strategies—inductions to drive legacy motives and inductions to encourage place attachment—can be useful in increasing adults’ intention to take action and their subsequent actions on climate issues. In the absence of age interactions, it appears that these strategies are equally effective across the lifespan. The potency of our findings imply their potential utility in promoting sustainability behaviors through legacy-based or place-based message framing or other forms of intervention, and so warrant further research.

Our theoretical model hypothesized separate pathways for the impacts of the legacy induction and place induction on legacy motivation and biophilia respectively. Although we did find that the Legacy induction was associated with significantly greater legacy motivation than the Place induction, we found higher legacy motivation and biophilia attitudes in both the place and legacy conditions, when compared to the control condition. This warrants further attention. It may be that our operationalization of the two inductions was not sufficiently differentiated (e.g., mention of “future generations” in the place induction may have elicited legacy concerns). It also may be that both inductions affected an underlying third element that itself affected both legacy motivation and biophilia. Future research should explore these possibilities, as well as the mechanism underlying why increased legacy motivation increases biophilia.

We had hypothesized that the Place induction would impact environmental hazard belief or environmental hazard concern: the belief in the general connection between natural hazards, climate change, and salient danger. However, there was no effect of either induction on hazard belief and concern. It is possible that if these items had been reworded to focus on the specific concern of the place to which participants were attached, the Place induction would have had a positive impact on hazard concern and belief. Additional research should continue to explore whether inductions that enhance one's connection to a place can be used to change risk attitudes related to natural hazards.

The current study has a number of strengths and limitations. One important limitation is that we did not have a direct manipulation check for place-based attachment—had we included this in addition to our biophilia measure, we could have more clearly and precisely discerned the relationships between these variables. The balanced age sampling allowed comparisons to be made across young, middle aged and older adults. However, the results are based on self-report from participants in a paid online survey, which may limit generalizability. Further research on the impact of these inductions on behavior in non-experimental settings would be useful. Additionally, the sample used in this research was limited to American participants. Future work could evaluate whether these findings generalize cross-culturally [41].

The time of measurement may also have unintentionally affected the patterns of results: data was gathered from participants in the month following the 2016 presidential election in the United States. President-elect Trump had already signaled his intention to leave the Paris climate accords and to roll back a variety of EPA regulations. Public discussion of these developments may have affected the participants’ responses. In addition, evaluations of the impact of combining moral framing [42] with legacy or place inductions would be useful. Finally, the outcome variables were limited in scope. Additional research in non-experimental settings could expand the outcome variables to sustainability behaviors such as recycling, changes in dietary and travel patterns, or changes in commuting behavior.

The world is experiencing two, simultaneous, global patterns: population aging and climate change. Despite generational differences in climate concerns [43], more than half of older adults are in the “worried middle”: they know something is happening to the world’s climate but they are unsure about what to do. The challenge is how to tap into this concern and move older adults from anxiety to pro-environmental action. Thus far, climate advocacy and message framing has not given adequate attention to older adults who have time, talent, and motivation to engage in issues beyond themselves. This study suggests that legacy and place message framing may prompt positive climate actions by adults of all ages. Those concerned with increasing individual and community responses to climate change must expand their age targets to include older adults, as well as younger and middle-aged adults. These results indicate that appeals to either their legacy concerns or their attachments to place can help move adults of all ages from anxiety to action.

## Supporting information

S1 File Methodological details. (DOCX)

S2 File Experimental induction essay prompts. (DOCX)

S3 File Supplementary parallel mediation analyses and correlation table. (DOCX)

S4 File Non-significant and supplementary results. (DOCX)
