# Supplementary material for: The impact of place and legacy framing on climate action: A lifespan approach
Source: PLoS One. 2020 Feb 25;15(2):e0228963. doi: 10.1371/journal.pone.0228963 (PMC7041806; doi:10.1371/journal.pone.0228963)
Supplement: S1 File — (DOCX) [file pone.0228963.s001.docx]

**S1 File. Methodological details.**

**Legacy Motivation Scale Question Items:**

Instructions: Please read each of the statements below carefully. Then **select the answer choice indicating the extent to which each statement describes you.** Please be as honest and accurate as you can be.

Scale: 1 (Not at all); 2 (Not very much); 3 (A little bit); 4 (Somewhat); 5 (A good deal); 6 (A great amount)

1. I care about what future generations think of me
2. I have important skills I can pass along to others
3. I am good at many things

4. I am well liked by my friends

5. Others would say that I have made unique contributions to my community or society

6. It is important to me to leave a positive legacy

7. I feel a sense of responsibility to future generations

8. I feel a connection to future generations

**Abbreviated Biophilia Scale Question Items:**

Instructions: Please indicate how strongly you agree or disagree with each statement:

Scale: 1 (Strongly disagree); 2 (Disagree); 3 (Somewhat disagree); 4 (Neither agree nor disagree); 5 (Somewhat agree); 6 (Agree); 7 (Strongly agree)

1. I feel joy just being in nature
2. Protecting the wellbeing of nature for its own sake is important to me
3. I feel a personal sense of interconnectedness with the rest of nature

4. I often feel a sense of awe and wonder when I am in unspoilt nature

5. I often feel a strong sense of care towards the natural environment

**Pro-Environmental Behavioral Intentions Scale Question Items:**

Instructions: Please indicate **how often you intend to perform** the following behaviors **over the next month:**

Scale: 1 (Never); 2 (Very Infrequently); 3 (Once in a while); 4 (Sometimes); 5 (Often); 6 (All the time)

1. Take showers that are 5 minutes or less

2. Use public transportation or carpool
3. Unplug appliances and chargers (e.g., TV, cell phone, computer) at night

4. Buy green products instead of regular products (e.g., dishwashing detergent), even though they cost more

5. Cut down on your carbon emissions (e.g., by reducing energy use)

6. Write letters, email, phone or otherwise contact elected official to urge them to take action on environmental issues (e.g., climate change, air pollution)

**Donations to an Environmental Charity Question Wording:**

Instructions: Before you finish our survey and as an extra "thank you" for participating in our research today, we will enter you into a lottery to win a $10 bonus. One study participant will be chosen at random to receive this bonus (which will be given to you via MTurk).

We also would like to give you an opportunity to donate some or all of the bonus to a charitable organization, if you are the lottery winner. You may split the $10 between yourself and the charity however you want to, using the form on the next page. Any money you allocate to the charity will be directly donated on your behalf by the research team.

The organization you have an opportunity to donate to today is called Trees for the Future, whose motto is "Plant trees. Change Lives." Since 1989, Trees for the Future has helped communities in 19 countries around the world plant millions of trees. Their work has and will continue to improve the well-being of children and families for generations to come, by cleaning the air, reducing risks from landslides and reducing deforestation. If you'd like to learn more about the organization, their website is: http://www.treesforthefuture.org


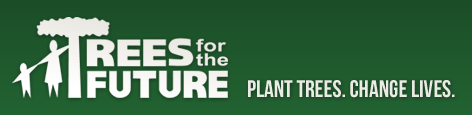


Please note that the total amount must add up to exactly $10. Remember that you will be paid your MTurk compensation regardless of whether you win the lottery or not.

Donate **____**

Keep for myself **____**

**Total ____**

**Natural Hazard Worry Question Wording:**

Instructions: How much do you personally worry about climate change’s effect on increasing natural hazard frequency and magnitude (such as tornadoes, hurricanes, floods, landslides)?

Scale: 1 (Not at all); 2 (Only a little); 3 (A fair amount); 4 (A great deal)

**Natural Hazard Belief Question Wording:**

Instructions: How convinced are you that climate change will have an effect on natural hazard frequency and magnitude? (such as tornadoes, hurricanes, floods, landslides)?

Scale: 1 (Not at all convinced); 2 (Not so convinced); 3 (Mostly convinced); 4 (Completely Convinced)

**Future Self-Continuity Question Wording:**

Instructions: How similar/connected do you feel to your future self 5 years from now? Please indicate which of the lettered pictures below best describes your relationship with your future self 5 years from now, where "A" indicates no connection and "G" indicates a very strong connection.

Scale:
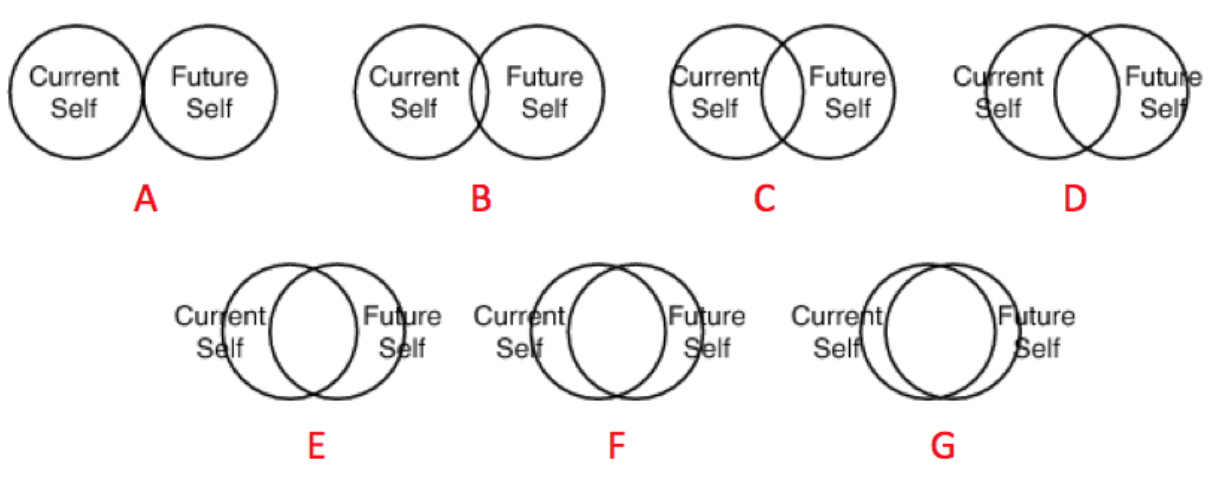


**Future Orientation Question Wording:**

Instructions: On a scale of 1 to 10, how easily does life in the year 2050 come to mind?

Scale: 1 (Not at all easily); 2; 3; 4; 5; 6; 7; 8; 9; 10 (Very easily)

**Internal Locus of Control Question Wording:**

Instructions: On a scale of 1 to 10, how much control do you have over your fate?

Scale: 1 (No Control); 2; 3; 4; 5; 6; 7; 8; 9; 10 (Full Control)

**Death Thought Accessibility Scale Items:**

Instructions: We are simply pre-testing this questionnaire for future studies. Please complete the following by filling letters in the blanks to create words. Please fill in the blanks with the first word that comes to mind. Write one letter per blank. Some words may be plural. Thank you.

1. BUR _ _ D

2. PLA _ _

3. _ _ OK

4. WAT _ _

5. DE _ _

6. MU _ _

7. _ _ N

8. B _ T _ LE

9. M _ J _ R

10. P _ _ TURE

11. FL _ W _ R

12. GRA _ _

13. K _ _ GS

14. CHA _ _

15. KI _ _ ED

16. CL _ _ K

17. TAB _ _

18. W _ _ DOW

19. SK _ _ L

20. TR _ _

21. P _ P _ R

22. COFF _ _

23. _ O _ SE

24. POST _ _

25. R _ DI _

**Demographics Questions:**

1. **What is your age (in years?)**
2. **What is your gender?**
3. **How much education have you completed? (Select from the following options)**
   1. **Some high school**
   2. **Graduated high school**
   3. **Some college or technical school**
   4. **Graduated college or technical school**
   5. **Post-graduate education**
4. **Generally speaking, do you usually think of yourself as a Republican, a Democrat, an Independent, or something else (Other)?**
5. **What is your race/ethnicity? (mark all that apply)**
   1. **Caucasian**
   2. **African American**
   3. **Hispanic**
   4. **Asian**
   5. **Native American**
   6. **Other**
6. **Do you consider yourself to be an environmentalist? (Yes/No)**

**
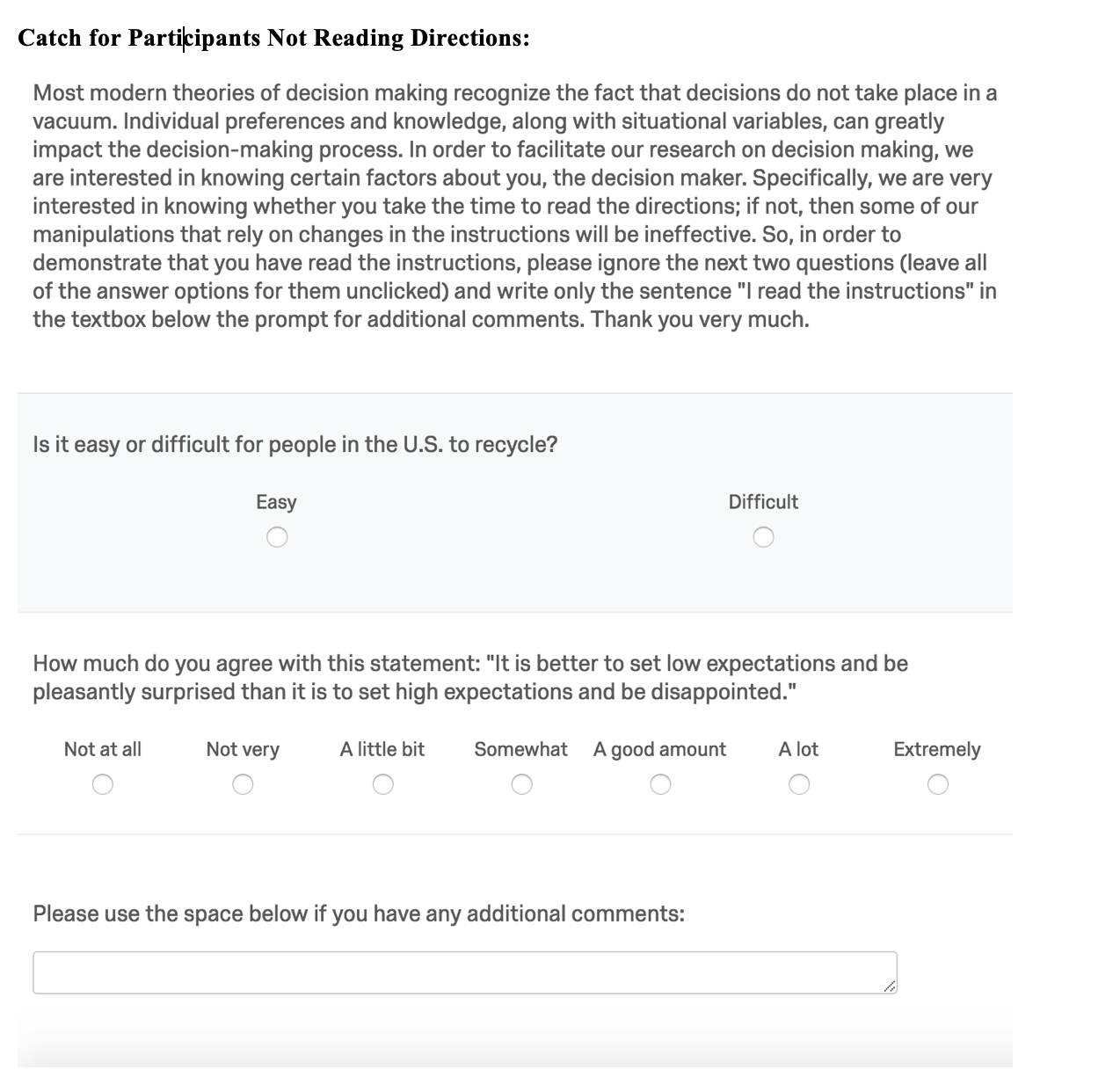
**
