# Supplementary material for: The impact of place and legacy framing on climate action: A lifespan approach
Source: PLoS One. 2020 Feb 25;15(2):e0228963. doi: 10.1371/journal.pone.0228963 (PMC7041806; doi:10.1371/journal.pone.0228963)
Supplement: S2 File — (DOCX) [file pone.0228963.s002.docx]

**S2 Text. Experimental induction essay prompts.**

**Experimental Place-Based Induction Essay Prompt:**

**Thinking about a place that holds special meaning for you**

For this writing task, we would like you to think about a place that you have an emotional connection to. Once you have a place in mind, can you imagine the impact of extreme weather events or climate change on that place? What would you like that place to be like for future generations after you’re gone? What positive things can you do to make sure that happens? In the space below, please write a brief essay describing your response to this set of questions and try to be as honest as you can be.

This essay should take you approximately 5-7 minutes to complete (roughly half a page).

**Experimental Legacy Induction Essay Prompt:**

**Thinking about your environmental legacy**

For this writing task, we would like you to think about what you want future generations to remember you for when you're gone. In answering this question, we'd like you to think about ways in which you will have a positive impact on the environment or on other people. For example, you might think about things you have done to reduce your personal use of natural resources like water and energy, whether you did all that you could do to minimize your emissions of air pollutants, or how strongly you worked to support laws and efforts to improve the environment. In the space below, please write a brief essay describing your response to this question and try to be as honest as you can be.

This essay should take you approximately 5-7 minutes to complete (roughly half a page).

**Control Induction Essay Prompt:**

**Thinking about how to scramble eggs**

Before moving on to the next questions, we would like you to participate in a short writing task to help clear your mind. Please describe in detail how to make scrambled eggs. Assume that you are writing directions for people who have not cooked scrambled eggs before, and outline for them a step-by-step process for making scrambled eggs.

This writing task should take you about 5-7 minutes to complete.
