# Supplementary material for: The impact of place and legacy framing on climate action: A lifespan approach
Source: PLoS One. 2020 Feb 25;15(2):e0228963. doi: 10.1371/journal.pone.0228963 (PMC7041806; doi:10.1371/journal.pone.0228963)
Supplement: S3 File — (DOCX) [file pone.0228963.s003.docx]

**S3 File. Supplementary parallel mediation analyses and correlation table.**

We conducted four additional parallel mediation PROCESS analyses including multiple mediators simultaneously to explore indirect effects while controlling for other mediators. In our first two analyses, we explored whether biophilia attitudes and legacy motives explained part of the relationship between the Legacy induction and behavioral intentions, and between the Legacy induction and donations.

Results from the first parallel mediation analysis indicated that exposure to the Legacy induction was indirectly related to behavioral intentions through its relationship with both legacy motivations and biophilia attitudes. First, those exposed to the Legacy induction reported greater biophilia (*a_1_* = .188, *p* = .009) and greater biophilia was subsequently related to greater behavioral intentions (*b_1_* = .321, *p* < .001). Similarly, those exposed to the Legacy induction reported greater legacy motives (*a_2_* = .348, *p* < .001) and greater legacy motives was subsequently related to greater behavioral intentions (*b_2_* = .308, *p* < .001). A 95% bias-corrected confidence interval based on 10,000 bootstrap samples indicated that the indirect effect through biophilia (a_1_b_1_ = 0.060, 95% CI = [0.016, 0.107]) and through legacy motives (a_2_b_2_ = 0.107, 95% CI = [0.065, 0.155]) was entirely above zero, holding all other mediators constant. Finally, those exposed to the legacy induction reported greater behavioral intentions even when taking into account the indirect effects of both the biophilia and legacy pathways (c’ = 0.135, *p* = .039)

Biophilia (M1)

*b_1_* = 0.321***

*a_1_* = 0.188**

Legacy (M2)

*b_2_* = 0.308***

*a_2_*= 0.348***

c’ = 0.135*

Behavioral Intentions (Y)

Legacy Induction (X)

(c = 0.303***)

**S1 Fig. Mediation of Legacy induction and behavioral intentions by biophilia attitudes and legacy motives.** Note: ****p* < .001. ***p* < .01. **p* < .05. ^#^ *p* = ns

Biophilia (M1)

*b_1_* = 0.729***

*a_1_* = 0.190**

Legacy (M2)

*b_2_* = 0.280*

*a_2_*= 0.346***

c’ = 0.035^#^

Donations (Y)

Legacy Induction (X)

(c = 0.270^#^)

**S2 Fig. Mediation of Legacy induction and donations by biophilia attitudes and legacy motives.** Note: ****p* < .001. ***p* < .01. **p* < .05. ^#^ *p* = ns

Results from the second parallel mediation analysis indicated that exposure to the Legacy induction was indirectly related to donations through its relationship with both legacy motivations and biophilia attitudes. First, those exposed to the Legacy induction reported greater biophilia (*a_1_* = .190, *p* = .008) and greater biophilia was subsequently related to more donations (*b_1_* = .729, *p* < .001). Similarly, those exposed to the Legacy induction reported greater legacy motives (*a_2_* = .346, *p* < .001) and greater legacy motives was subsequently related to more donations (*b_2_* = .280, *p* = .019). A 95% bias-corrected confidence interval based on 10,000 bootstrap samples indicated that the indirect effect through biophilia (a_1_b_1_ = 0.138, 95% CI = [0.038, 0.245]) and through legacy motives (a_2_b_2_ = 0.097, 95% CI = [0.014, 0.198]) was entirely above zero, holding all other mediators constant. After taking into account the indirect effects of both the biophilia and legacy pathways, there was no direct relationship between the Legacy induction and donations (c’ = 0.035, *p* = .869).

In our second two analyses, we explored whether biophilia attitudes and legacy motives explained part of the relationship between the Place induction and donations, and between the Place induction and behavioral intentions. Results from the first parallel mediation analysis indicated that exposure to the Place induction had a marginally significant relationship with biophilia (*a_1_* = .124, *p* = .082) and no significant relationship with legacy motivation (*a_2_* = .032, *p* = .605). However, greater biophilia was associated with more donations (*b_1_* = .721, *p* < .001). Similarly, greater legacy motives were associated with more donations (*b_2_* = .284, *p* = .016). A 95% bias-corrected confidence interval based on 10,000 bootstrap samples suggested a lack of indirect effects. Finally, after taking into account both biophilia and legacy motives, there was no direct relationship between the Place induction and donations (c’ = 0.319, *p* = .127).

Biophilia (M1)

*b_1_* = 0.721***

*a_1_* = 0.124^#^

Legacy (M2)

*b_2_* = 0.284*

*a_2_*= 0.032^#^

c’ = 0.319^#^

Donations (Y)

Place Induction (X)

(c = 0.417^#^)

**S3 Fig. Mediation of Place induction and donations by biophilia attitudes and legacy motives.** Note: ****p* < .001. ***p* < .01. **p* < .05. ^#^ *p* = ns

Biophilia (M1)

*b_1_* = 0.321***

*a_1_* = 0.120^#^

Legacy (M2)

*b_2_* = 0.320***

*a_2_*= 0.043^#^

c’ = 0.050^#^

Behavioral Intentions (Y)

Place Induction (X)

(c = 0.102^#^)

**S4 Fig. Mediation of Place induction and behavioral intentions by biophilia attitudes and legacy motives.** Note: ****p* < .001. ***p* < .01. **p* < .05. ^#^ *p* = ns

Results from the final parallel mediation analysis indicated that exposure to the Place induction had a marginally significant relationship with biophilia (*a_1_* = .120, *p* = .095) and no relationship with legacy motivation (*a_2_* = .043, *p* = .494). However, greater biophilia was associated with greater behavioral intentions (*b_1_* = .321, *p* < .001). Similarly, greater legacy motives were associated with greater behavioral intentions (*b_2_* = .320, *p* < .001). A 95% bias-corrected confidence interval based on 10,000 bootstrap samples suggested a lack of indirect effects. Finally, after taking into account both biophilia and legacy motives, there was no direct relationship between the Place induction and donations (c’ = 0.319, *p* = .127).

| **Table S1. Pearson Correlations Among Variables of Interest** | | | | |  |  |  |
| --- | --- | --- | --- | --- | --- | --- | --- |
|  |  | 1. Legacy motivation total | 2.biophilia total | 3.behavioral intent total | 4.Donate to Trees for the Future | 5.Dum_Legacy: Received Legacy Induction | 6. Dum_Place: Received Place Induction |
| 1. Legacy motivation total | Pearson Correlation | 1 |  |  |  |  |  |
| 2. biophilia total | Pearson Correlation | .396^**^ | 1 |  |  |  |  |
| 3. behavioral intent total | Pearson Correlation | .398^**^ | .423^**^ | 1 |  |  |  |
| 4. Donate to Trees for the Future | Pearson Correlation | .175^**^ | .276^**^ | .242^**^ | 1 |  |  |
| 5.Dum_Legacy: Received Legacy Induction | Pearson Correlation | .178^**^ | .083^**^ | .129^**^ | .036 | 1 |  |
| 6. Dum_Place: Received Place Induction | Pearson Correlation | .017 | .050 | .045 | .063^*^ | -.430^**^ | 1 |
| **. Correlation is significant at the 0.01 level (2-tailed). | | | | | | | |
| *. Correlation is significant at the 0.05 level (2-tailed). | | | | | | | |
