# Supplementary material for: The impact of place and legacy framing on climate action: A lifespan approach
Source: PLoS One. 2020 Feb 25;15(2):e0228963. doi: 10.1371/journal.pone.0228963 (PMC7041806; doi:10.1371/journal.pone.0228963)
Supplement: S4 File — (DOCX) [file pone.0228963.s004.docx]

**S4 Text. Non-significant and supplementary results.**

**Death Thought Accessibility Scale.** Neither induction (*p* = .725) nor age (*p* = .392) were significantly predictive of Death Thought Accessibility, suggesting that experimental conditions had no significant impact on mortality salience.

**Future-Self Continuity (FSC).** No significant main effect emerged for induction on Future-Self Continuity (*p* = .201) but a significant main effect emerged for age *F* (2, 978) = 39.95, *p* < .001, *h_p_^2^* *=* .076, such that older adults had significantly more FSC than middle-aged adults, and middle-aged adults had significantly more FSC than young adults. A significant interaction between induction and age emerged *F* (4, 978) = 2.38, *p* = .050, *h_p_^2^* *=* .010. Age was revealed to moderate the impact of the Legacy induction on FSC, such that being an older adult who was exposed to the legacy induction was associated with significantly higher levels of FSC than in any other condition.

**Future Orientation Measure.** A significant main effect emerged for induction *F* (2, 979) = 3.29, *p* = .038, *h_p_^2^* *=* .007, such that the place induction was associated with more future orientation than the legacy (*p* = .036) or control (*p* = .028) inductions, while the legacy and control inductions did not differ significantly from each other. A significant main effect also emerged for age on future orientation *F* (2, 979) = 4.06, *p* = .018, *h_p_^2^* *=* .008, such that older adults had significantly less future orientation than their middle-aged adults or younger counterparts, while middle-aged and young adults did not differ significantly. No significant interaction effect between age and induction on future orientation emerged (*p* = .897).

**Locus of Control.** A significant main effect emerged for induction *F* (2, 978) = 4.56, *p* = .011, *h_p_^2^* *=* .009, such that the place induction (*p* = .002) was associated with a more external locus of control than the legacy induction, but did not differ significantly from the control induction. Age did not significantly predict Locus of Control (*p* = .228), nor did an interaction effect emerge (*p* = .897).

**Natural Hazard Belief.**  Induction did not significantly predict Natural Hazard Belief (*p* = .399), and while no interaction effect emerged (*p* = .583), age was marginally predictive of Natural Hazard Belief (*p* = .063), such that young adults had less belief in natural hazards than middle-aged or older adults.

**Natural Hazard Worry.** Neither induction (*p* = .092) nor age (*p* = .225) were significantly predictive of Natural Hazard Worry, nor was an interaction observed (*p* = .603), suggesting that experimental conditions had no significant impact on participant concerns regarding natural hazards.
